# Quantitative biomedical annotation using medical subject heading over-representation profiles (MeSHOPs)

**DOI:** 10.1186/1471-2105-13-249

**Published:** 2012-09-27

**Authors:** Warren A Cheung, BF Francis Ouellette, Wyeth W Wasserman

**Affiliations:** 1 Centre for Molecular Medicine and Therapeutics at the Child and Family Research Institute, Department of Medical Genetics, University of British Columbia, Vancouver, BC, Canada; 2 Bioinformatics Graduate Program, University of British Columbia, Vancouver, BC, Canada; 3 Ontario Institute for Cancer Research, Toronto, ON, Canada; 4 Department of Cell and Systems Biology, University of Toronto, Toronto, ON, Canada

## Abstract

**Background:**

MEDLINE®/PubMed® indexes over 20 million biomedical articles, providing curated annotation of its contents using a controlled vocabulary known as Medical Subject Headings (MeSH). The MeSH vocabulary, developed over 50+ years, provides a broad coverage of topics across biomedical research. Distilling the essential biomedical themes for a topic of interest from the relevant literature is important to both understand the importance of related concepts and discover new relationships.

**Results:**

We introduce a novel method for determining enriched curator-assigned MeSH annotations in a set of papers associated to a topic, such as a gene, an author or a disease. We generate MeSH Over-representation Profiles (MeSHOPs) to quantitatively summarize the annotations in a form convenient for further computational analysis and visualization. Based on a hypergeometric distribution of assigned terms, MeSHOPs statistically account for the prevalence of the associated biomedical annotation while highlighting unusually prevalent terms based on a specified background. MeSHOPs can be visualized using word clouds, providing a succinct quantitative graphical representation of the relative importance of terms. Using the publication dates of articles, MeSHOPs track changing patterns of annotation over time. Since MeSHOPs are quantitative vectors, MeSHOPs can be compared using standard techniques such as hierarchical clustering. The reliability of MeSHOP annotations is assessed based on the capacity to re-derive the subset of the Gene Ontology annotations with equivalent MeSH terms.

**Conclusions:**

MeSHOPs allows quantitative measurement of the degree of association between any entity and the annotated medical concepts, based directly on relevant primary literature. Comparison of MeSHOPs allows entities to be related based on shared medical themes in their literature. A web interface is provided for generating and visualizing MeSHOPs.

## Background

The MEDLINE®/PubMed® bibliographic database of the U.S. National Library of Medicine (NLM) is an actively maintained central repository of over 18.5 million biomedical literature references [1]. To navigate this growing body of published information, the MEDLINE®/PubMed® references are indexed by subject experts at the NLM using Medical Subject Headings (MeSH) [2], a structured controlled vocabulary of 26,000 biomedical descriptors. The MeSH annotations are intended to facilitate the identification of relevant papers for research scientists. As MEDLINE®/PubMed® grows at a modern rate exceeding 600,000 references per year, researchers face a daunting challenge to assess the body of work about entities (genes, drugs, authors, etc.) arising in the course of their research.

Encapsulating the bibliography for a biomedical entity of interest in a form both understandable and informative is an increasingly important challenge in biomedical informatics [3,4]. One approach to succinctly summarise a bibliography (e.g. a set of key papers) for a biomedical topic is to identify the MeSH terms most strongly associated to the papers. Previous reports which introduced summaries of over-represented MeSH terms for a set of papers include a study of enriched annotations for groups of differentially expressed genes [5] and a method to identify MeSH terms enriched in articles retrieved in a query of the PubMed database [6]. These initial approaches to MeSH annotation analysis applied *ad hoc* measures of association over small sets of articles to demonstrate the potential value for MeSH annotation summarization.

Key to accelerating the research process is the development of systematic approaches to quantitatively represent bibliometric information and infer functionally important relationships between entities. Addressing this goal, we introduce MeSH Over-representation Profiles (MeSHOPs) to quantitatively describe the properties of genes, diseases or any other entity associated with a set of articles represented in MEDLINE®/PubMed®. The entire MEDLINE®/PubMed® database (hereafter referred to as MEDLINE) is analyzed. For each MeSHOP, the over-representation of MeSH annotations across a bibliography of articles is statistically evaluated for a biomedical topic. MeSHOPs convey characteristics of the subject entity, facilitating discovery of novel relationships across classes of entities. We demonstrate the use of MeSHOPs to facilitate property visualization, subject to the use of appropriate corrections for background annotation properties. To assess the utility of MeSHOPs for high-throughput generation of quantitative annotation, the capacity of the process to re-derive a subset of Gene Ontology annotation of genes is measured. Using a class of biomedical entities – vitamins – as an example, MeSHOPs comparisons are shown to provide a quantitative measure of similarity between each member of the class. Profiles can be similarly compared across entity classes, as demonstrated in an analysis of the similarities between gene MeSHOPs and brain disease MeSHOPs. MeSH Over-representations Profiles fill an important niche in computational biology, allowing quantitative annotation descriptions to be generated for any entity for which a set of research articles indexed in the MEDLINE database can be defined.

## Methods

### Calculating MeSH over-representation profiles

**Figure 1:**
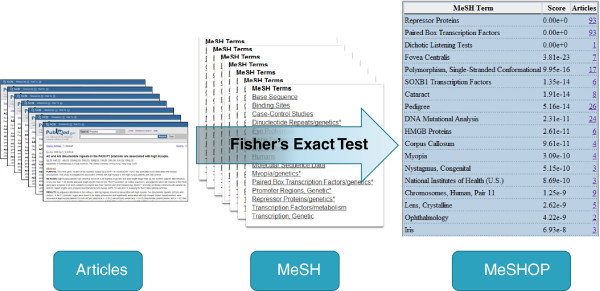
**Workflow for Generating a MeSHOP.** Starting from a set of articles relating to a biological concept or entity (the foreground set), the associated MeSH terms for each PubMed record of each article are extracted. The prevalence of each MeSH term across the set of articles is compared to a background. Fisher’s Exact Test is applied to measure the statistical over-representation of each term in the foreground set.

A MeSHOP is a quantitative representation of the annotations associated with a set of articles, where the set is composed of articles that address a specific entity (such as a gene or disease). The computation of a MeSHOP initiates from a set of articles that address a specific entity and returns a set of over-represented MeSH terms, each term with a *p*-value reflecting over-representation based on its rate of occurrence in the set of articles (see Figure 1). Comparing the observed frequency of each MeSH term annotated to the background rate returns a measure of over-representation. A MeSHOP is a vector of tuples < (*t*_*1*_*, m*_*1*_), (*t*_*2*_*, m*_*2*_), … (*t*_*n*_*, m*_*n*_) >. For each tuple (*t*_*i*_*, m*_*i*_) in a MeSHOP, *t*_*i*_ is a distinct MeSH term in the MeSH vocabulary and *m*_*i*_ is the numeric measure of the over-representation of MeSH term *t*_*i*_ to the set of articles. For this study, several large classes of entities were analyzed such as the human *genes* in Entrez Gene and the *diseases* specified formally within MeSH.

MeSHOPs are generated for each member of a class by assessing the set of all linked MEDLINE records for each member. We use Fisher’s Exact Test to determine *p*-values, computed from a 2x2 contingency table comprised of: 1) the frequency of occurrence of the term *t*_*i*_ in the set of articles addressing the entity of interest; 2) all articles addressing the entity of interest without the term *t*_*i*_; 3) the frequency of the term *t*_*i*_ in the background set not addressing the entity of interest; and 4) the remaining number of articles in the background set that do not refer to the term *t*_*i*_. and do not address the entity of interest. The universal background studied is the set of 17 million MEDLINE articles assigned MeSH terms, with class-specific background comprising a subset of these articles (see Additional file 1: Table S2 for more details).

### Annotation data

Over 18 million biomedical references in MEDLINE have been evaluated by NLM staff subject experts. These curators assigned appropriate MeSH terms corresponding to the topics covered by the paper. The MeSH terms chosen are intended to be the most specific terms relevant to the topic covered in the paper – for example, if the term “Alzheimer Disease” is attached to the paper, the more general (‘parental’) term “Brain Disease” would not be associated. For our analysis, we therefore consider a paper annotated by a MeSH term to also be annotated with all ‘parents’ (and ‘grand-parents’, and ‘great grand-parents’, and so on to the root of the hierarchy) of that MeSH term. When indexing articles using MeSH terms, the scope of a complex topic often cannot be covered using a single MeSH term. In this case, multiple separate terms are “coordinated”. For example, the topic “medical staff in teaching hospitals” is covered by annotating with the two separate distinct MeSH terms “Medical Staff, Hospitals” and “Hospitals, Teaching”. There is no indication that the two terms are linked within the record.

### Generating disease MeSHOPs

For each MeSH term from the disease category (Category C), the entire bibliography of annotated articles in MEDLINE was considered. Disease-article linkages are drawn directly from MEDLINE via the curator-assigned MeSH terms. To generate MeSH term literature profiles for diseases, all MeSH terms from the disease category – Category C – were used; a set composed of 4 494 terms in MeSH 2011 linking to over 10 million articles.

### Generating gene MeSHOPs

All human genes in Entrez Gene were considered (45 333 in Entrez Gene 2011). Two sources for gene-article linkages from Entrez Gene were evaluated: Gene Reference Into Function (GeneRIF, http://goo.gl/SzRui) and *gene2pubmed* (http://goo.gl/bUEDU). GeneRIF is a curated set of links provided by annotators at the NLM and public submissions, where each set of PubMed articles refers to a briefly described function of the gene. *gene2pubmed* is a set of links to PubMed articles relating to the gene, generally broader in scope than GeneRIFs. GeneRIFs link 15 312 human genes to 213 595 articles. *gene2pubmed* links 30 324 human genes to 302 629 articles.

### Generating chemical compound MeSHOPs

We examine all chemical compounds annotated to MEDLINE articles. These include chemical compounds that are part of the main MeSH hierarchy (Category D), as well as chemical compounds that are part of the Supplementary Concept Records.

### Generating MesHOP word clouds

The MeSHOP [*term*, -log(*p-value*)] pairs are submitted to the online cloud generating software Wordle (http://www.wordle.net) and visualized using the “Horizontal” layout. We cap the minimum for the p-values at 10^-30^. Each MeSH term for a given MeSHOP is laid out in a random, non-overlapping manner, with the font size of the term scaled proportional to the weight in the vector. Users can generate a word cloud from a MeSHOP via a single click on the results page, or by copying and pasting the MeSHOP *term*-*value* pairs into the Wordle Advanced submission page.

### Implementation

The analysis was performed using Python (http://www.python.org/), XSLT (http://www.w3.org/TR/xslt), and the MySQL database system (http://www.mysql.com/). Fisher’s Exact Test *p*-values and hierarchical clustering using complete linkage and Euclidean distance were computed using the R statistics package (http://www.r-project.org/). Results were generated using 50 CPUs of a compute cluster running under Sun GridEngine (http://gridengine.sunsource.net/). A typical cluster machine is a 64-bit dual processor 3 GHz Intel Xeon with 16 GB of RAM.

Datasets were downloaded from Entrez Gene (ftp://ftp.ncbi.nlm.nih.gov/gene/). We analyze the 2011 MeSH-annotated MEDLINE®/PubMed® baseline (http://www.nlm.nih.gov/databases/leased.html). See Additional file 1: Table S1 for details of the size and contents of the datasets.

### Web Interface for generating and obtaining MeSHOPs

To enable reader exploration of the profiles, we provide pre-computed MeSHOPs for biomedical entities such as genes, diseases and chemical compounds (http://meshop.oicr.on.ca). All MeSH-annotated articles available through the 2011 full year release are incorporated into the profiles. Diseases include all specified by MeSH terms under the parent term “Diseases”. Chemical compounds are all compounds appearing in the MeSH supplemental concepts. Genes are not consistently defined as MeSH terms. As MeSHOPs may be generated for any set of articles, gene MeSHOPs were derived from existing mappings of genes onto PubMed article identifiers For the pre-computed datasets (genes, diseases and chemical compounds), we also provide the number of number of entities(e.g *n =* 4411 diseases), allowing users to use this to calculate Bonferroni-corrected *p*-values if they desire. Users seeking to generate MeSHOPs for other biomedical entities – for example, using entity-article mappings from another resource – can use the results of a PubMed search query or directly provide a list of PubMed Identifiers (PMIDs) to compute MeSHOPs.

## Results

MeSHOPs quantitatively represent the association of medical terms to a topic of interest, based on the bibliography for the topic compared to a background set of articles. We examine methods for generating MeSHOPs, and show how MeSHOPs can be used to reveal terms associated with a topic.

### MeSHOPs for biomedical entities

**Figure 2:**
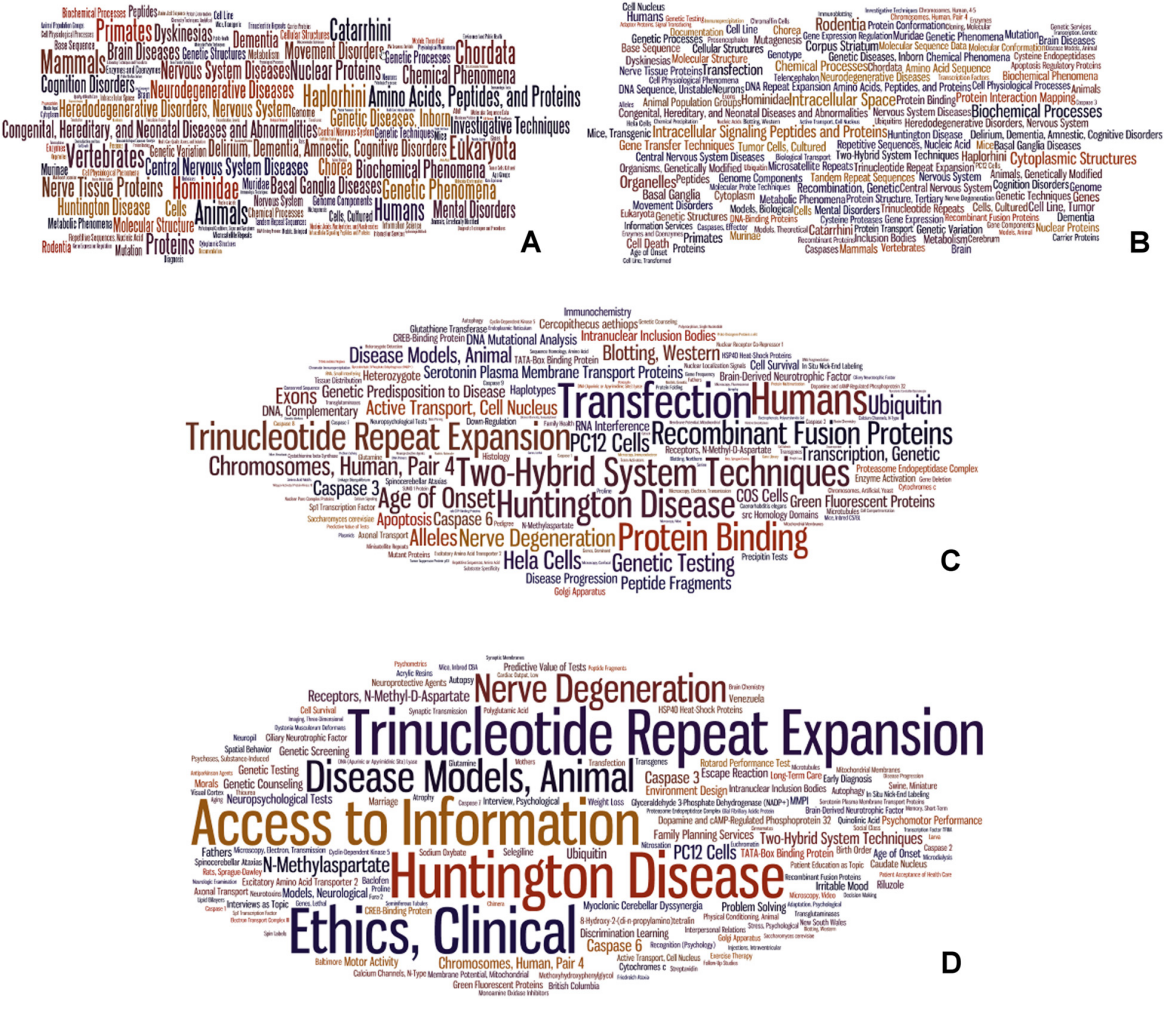
**Alternative Approaches for Generating MeSHOPs depicted as Word Clouds.** All MeSHOPs depict annotation of the HTT gene that is causal for Huntington Disease. (**A**) Raw counts. (**B**) Statistical enrichment scores. The top 150 terms in the profile are shown with the font size of the term is proportional to the negative log *p*-value for the term. Note the presence of many general terms which are implied by more specific terms, such as “Vertebrates”, “Primates”, “Chordata” and “Mammals” being present, but covered by the term “Humans”. Also, when studying a set of human genes, the terms “Humans” and “Genes” are commonly occurring and should be down-weighted accordingly. (**C**) Redundancy Filtered HTT Gene Biomedical Term Word Cloud. This is a word cloud where the more general terms have been filtered out from (**B**), leaving only the most specific terms in the profile. For example, the term “Repetitive Sequences, Nucleic Acid” seen in (**B**) has been filtered out due to the presence of the term “Trinucleotide Repeat Expansion”. (**D**) Redundancy Filtered HTT Gene Biomedical Term Word Cloud using human gene background. This is a word cloud when taking only the subset of PubMed articles related to human genes as the background, while also applying the filtering seen in (**C**).

To quantitatively describe the annotation properties of a biomedical entity using MeSH terms attached to a set of articles about the entity, we evaluated multiple procedures. At the simplest, one could count the number of times each MeSH term is attached to the corpus of articles (Figure 2A). Such an approach fails to account for the number of articles in the corpus, so one could normalize the frequency. While such a correction may facilitate comparisons between distinct MeSHOPs, it fails to account for the importance of the individual terms and has no impact on the visual representation (data not shown). Some terms, such as ‘human’ are attached frequently, but provide little information to distinguish between distinct biomedical entities. To place the quantitative emphasis on distinguishing terms, we elect to calculate a *p*-value reflecting the significance of observing the number of annotations with a MeSH term in a set of articles of the given corpus size (Figure 2B). The statistical model balances the number of articles related to the entity being profiled against the prevalence of the term in the background, providing greater emphasis on the occurrence of rare terms. The *p*-value computed therefore controls for the number of articles associated to an entity, against the null hypothesis of independent random assignment of MeSH terms to the articles related to the entity. This MeSHOP generation process (Figure 1) underlies all subsequent analysis in this report.

### Simplifying large MeSHOPs

**Figure 3:**
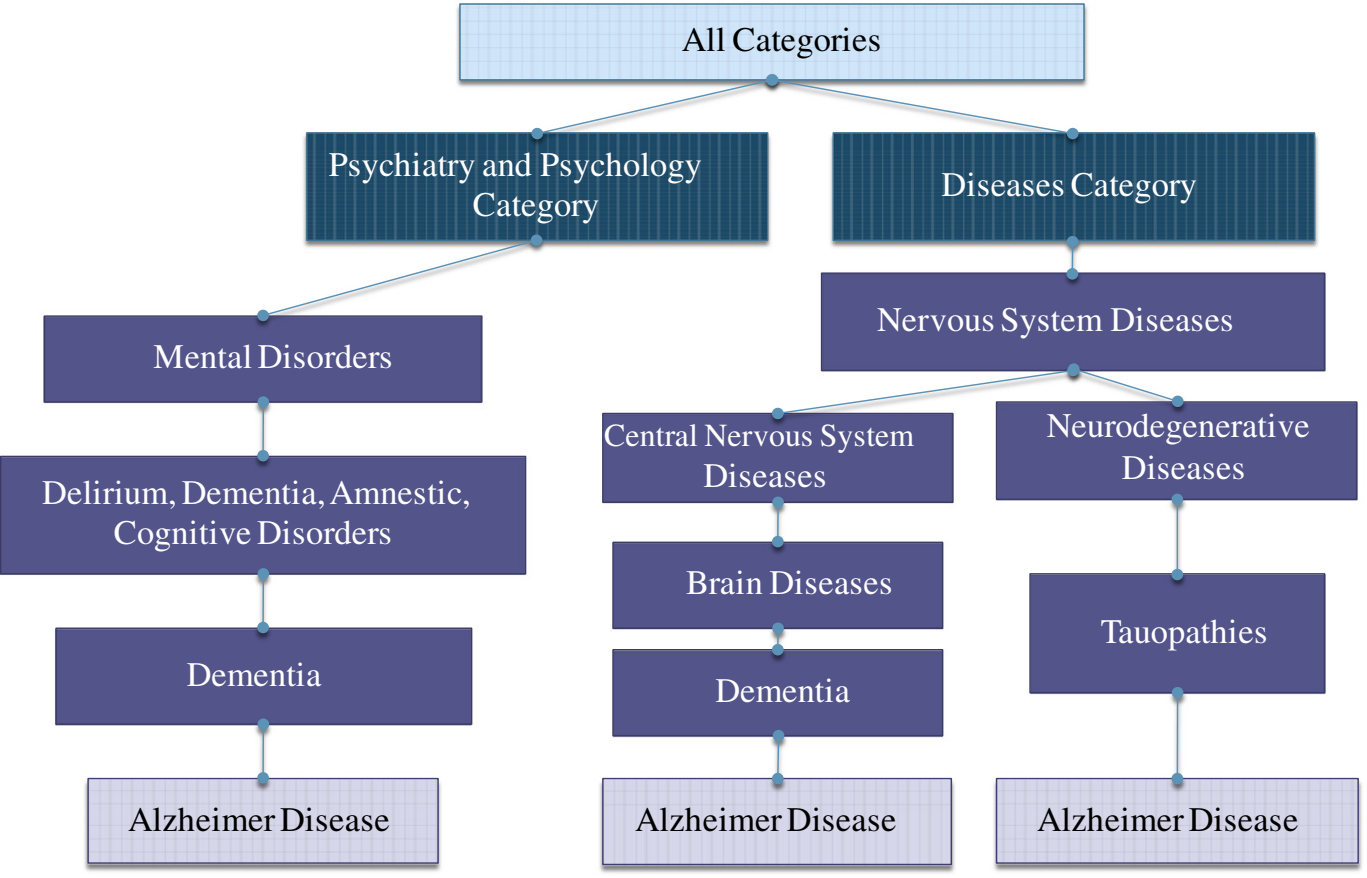
**Subset of the MeSH Tree for Alzheimer Disease.** The entries in the Medical Subject Heading tree leading to Alzheimer disease. Note that the term Alzheimer Disease occurs in three places in the tree, and under two separate subheadings in the Disease category – once under “Central Nervous System Diseases” due to its location in the human body, and once under “Neurodegenerative Diseases” and “Tauopathies” due to the type of disease.

Inspecting the raw MeSHOPs revealed two issues that become increasingly important when analyzing larger bibliographies: (i) highly correlated terms within the MeSH hierarchy result in concept redundancy in the profiles; and (ii) the universal background rate of term frequency results in uninformative class-enriched terms. Two corrections were introduced to address these issues. As an example of the first problem, consider the term “Alzheimer Disease”, which implies the more general term “Brain Disease”, rendering the observed over-representation of “Brain Disease” uninformative in a profile (see Figure 3). The tree-like structure of the MeSH vocabulary provides a direct method to determine term relationships. A more succinct representation can be generated by removing more general terms, limiting MeSHOPs to include only the most specific significantly associated terms from the MeSH tree (See Figure 2C). As an example of the second problem, the initial MeSHOP for the gene BRCA1 includes the term “polymorphism, single nucleotide”, however this term is enriched for 29% of human genes using the universal background set of articles. To address this issue, we calculate the enrichment statistics based on class-specific article backgrounds. For human genes, the background is restricted to articles addressing at least one human gene. Similarly, for diseases, the background is all articles annotated with at least one MeSH disease term. Using class-specific backgrounds, the statistical test highlights terms unusually enriched for the specific member, de-emphasizing terms common to all members of the class (see Figure 2D).

### Visualizing MeSHOPs

MeSHOPs can be directly converted into word clouds to provide a convenient graphical depiction of the annotation properties that enables rapid visual comparison of the relative importance of terms (See Figure 2). Word clouds for the MeSHOPs provide a visual representation of a MeSHOP, allowing for immediate evaluation of the most important terms as well as their relative importance, in a manner similar to sequence logos [7]. We have introduced above two approaches that improve over-representation profiles: (i) filtering to retain only the most specific MeSH terms and (ii) selecting an appropriate background for the statistical comparisons. A word cloud for a MeSHOP is generated using the associated MeSH terms and the negative log of the corresponding calculated *p*-values, directly translating the statistical significance of each term proportionally into the size of the font for the associated term.

### Properties of gene and disease annotation

**Figure 4:**
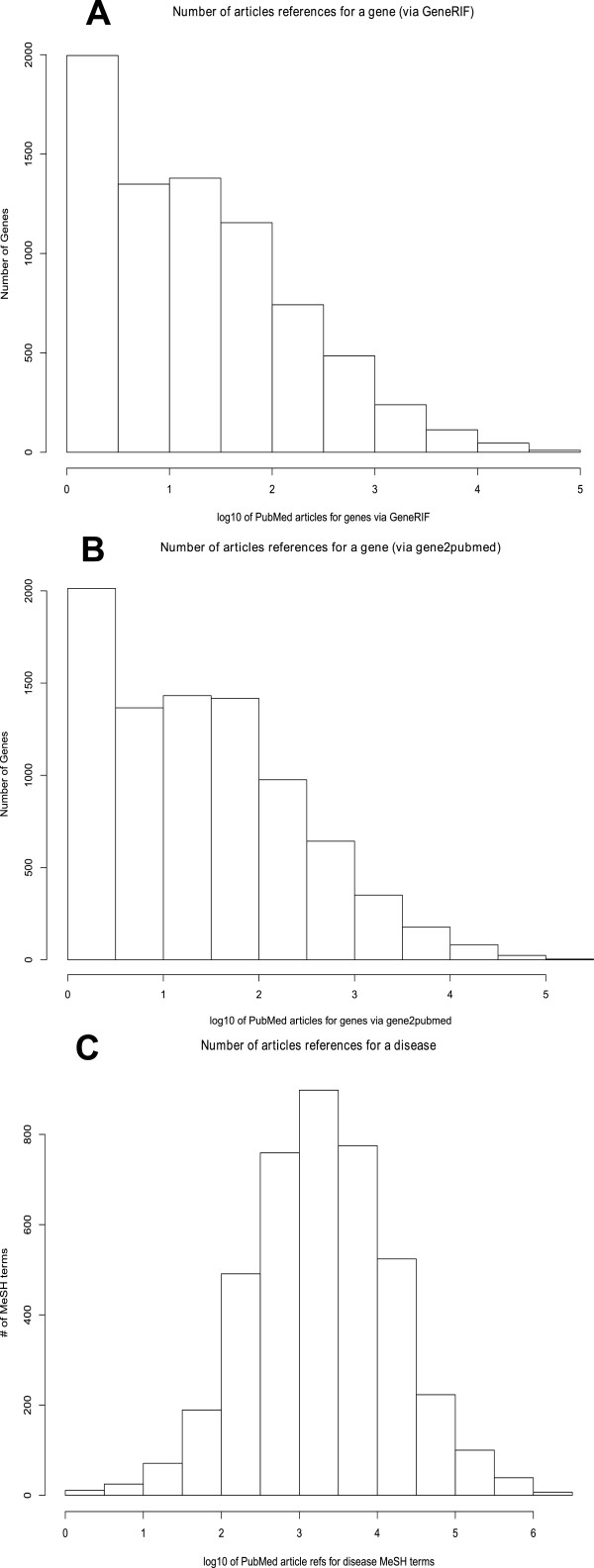
**(A) Distribution of Genes by Number of Associated GeneRIF References.** The distribution shows that the bulk of the genes have very few references, with an extreme tail of a small fraction of genes having a very large number of references. (**B**) Distribution of Genes by Number of Associated *gene2pubmed* References. Although overall average number of references is higher due to the larger number of *gene2pubmed* references, the distribution remains is very similar to (**A**). (**C**) Distribution of Diseases by Number of Associated PubMed References. Unlike the distributions of gene references, disease MeSH terms have substantial literature support, although there remains an extreme tail of a small fraction of MeSH terms having an extremely large number of articles.

Examination of the number of articles linked to human genes and diseases reveal substantial differences between these data sources. Most genes bibliographies have few linked articles, the distribution decreasing with an extreme tail of well-studied genes with many links. For the GeneRIF article links from Entrez Gene (accessed 2007-02-13), genes have a mean of 369 assigned articles, but a median of only 15 articles (See Figure 4A). Similarly, for the *gene2pubmed* article links, the mean is 637 articles, yet the median is only 20 articles (See Figure 4B). Diseases have a more balanced distribution, but still a characteristic extreme tail with of certain well-studied articles, with the distinct difference that very few diseases have only a couple articles. In the 2007 release of PubMed, a mean of 19 431 articles linked to each disease but a median of only 1 912 articles – still substantially more than the median for genes (See Figure 4C). Of the 24 357 MeSH 2007 terms, 15 674 terms are represented in gene MeSHOPs (via the 2007 *gene2pubmed* article links), and 23 473 terms are found in disease MeSHOPs (via 2007 PubMed). We expect that as genes become better annotated with more comprehensive bibliographies, their annotation pattern will come to resemble that of the more comprehensively annotated diseases.

### Re-deriving gene ontology annotations with MeSHOPs

MeSHOPs may be most advantageous as an approach to generate quantitative annotation profiles in a high-throughput manner for any set of biomedical entities that can be associated with sets of research articles. To measure of the performance of the procedure to regenerate relevant annotations, we assessed the sensitivity of MeSHOPs for detecting the directly mappable subset of Gene Ontology terms annotated to genes. Using the Unified Medical Language System (UMLS) mapping of MeSH terms to Gene Ontology terms, we identified 396 GO terms with one-to-one equivalent MeSH terms. Depicted in Additional file 1: figure S1A, we observe that the sensitivity of MeSHOPs for representing these terms for the corresponding genes ranges from 77% (at a *p*-value threshold of 0.05) to 95% (at a threshold of 0.31). As GO annotations are not comprehensive, there is no direct means to assess the specificity of the method. In lieu of specificity we plot the total number of MeSH terms mapped per gene relative to the threshold values, with 162 terms per gene at a *p*-value threshold of 0.05 (Additional file 1: Figure S1B).

### Temporal changes of MeSHOPs

**Figure 5:**
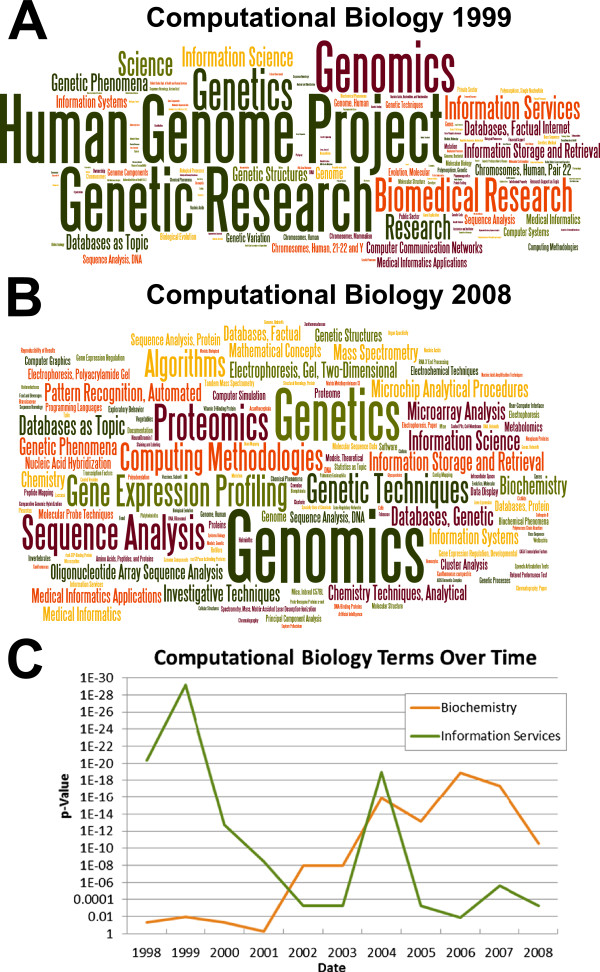
**MeSHOP for “Computational Biology”.** MeSHOPs were generated for the 50 most recent articles annotated with the MeSH term “Computational Biology” from the year 1999 (**A**) and the year 2008 (**B**). MeSHOPs were computed using the universal background from PubMed Baseline 2011 (covering articles through 2010). The MeSH term for “Computational Biology” and its parent terms were excluded from the MeSHOP. (**C**) Change in Significance of Biomedical Terms for Computational Biology over Time. The p-values for the terms “Biochemistry” and “Information Services” and their association to “Computational Biology” over time. For each time point, a MeSHOP using the most recent 50 articles for that year was generated to obtain the p-values for the terms.

MeSHOPs can be used to identify changing knowledge and properties for an entity. For example, by taking a subset of the articles for a biomedical entity at different timepoints, we can track the changes in research focus for the entity over time. Two areas of research, defined by the MeSH terms “Computational Biology” and “Stem Cells” were analyzed. At each selected time point, the fifty most recent articles for that year were taken to represent the state of the field at that time, and MeSHOPs were computed using the universal MEDLINE background. Analyzing the MeSHOPs for “Computational Biology” over the past decade allows us to quantitatively evaluate the evolution of the field (see Figure 5). For this analysis, all years indicate the inclusion of articles to the end of that calendar year. The MeSHOP from 1999 reveals significant topics such as “Human Genome Project”, a major informatics focus at that time point, that are completely absent when we examine the corresponding MeSHOP from 2008. “Genetic Research”, present in both MeSHOPs, is followed in the recent MeSHOP with other terms for biological disciplines and techniques such as “Genomics”, “Genetic Techniques”, “Proteomics” and “Sequence Analysis, Protein”, demonstrating how computational biology techniques are being more tightly integrated with biomedical research (see Additional file 1: Table S3). As seen in Figure 5C, data from MeSHOPs can be used to chart the gradual decline in significance of “Information Services” as the focus of the research switches from storage of the data, and the corresponding rise in association to “Biochemistry” demonstrating it more tightly coupling with scientific study. Similarly, we can track the changes in “Stem Cells” since the introduction of the term in 1984 (see Additional file 1: Figure S2). By 1985, we see “Hematopoietic Stem Cells” and “Bone Marrow Cells” as a significant focus. This is followed by the surge in importance of “Stem Cell Transplantation” by 2000, whereas by 2009 we see the focus shifting to “Mesenchymal Stem Cells”, “Cell Differentiation” and “Embryonic Stem Cells”.

MeSHOPs provide both a qualitative visual summary of the shifting focus of research over time for an entity of interest, as well as provide a method to quantitatively track the progression of association of biomedical subjects as they relate to the entity of interest.

### Intra-group MeSHOP similarity

MeSHOPs can also be used to investigate relationships between a set of related entities. For the set of entities comprising the 13 human Vitamins, we first use MeSHOPs to examine the co-occurrence of Vitamin MeSH terms in MEDLINE (See Additional file 1: Figure S3A) by considering, for each vitamin entity, the subset of the MeSHOP relating to vitamins. In this case, the MeSHOPs provide a measure of co-occurrence strength between any two vitamins, allowing us to visualize and cluster the vitamins via their bibliographic topic co-occurrence. We see the vitamins separating with the fat-soluble vitamins A,D,E and K together, whereas the water soluble vitamins (Ascorbic Acid and the B complex vitamins) are grouped separately. This graphic also reveals publication trends – for example, of the fat-soluble vitamins, all co-occur except for vitamins A and K, and the water-soluble vitamins clustering into three distinct groups, with Niacin separated from Pantothenic Acid, Biotin and Thiamine, which are also separate from the rest of the B complex vitamins which group with Ascorbic Acid.

Using the entirety of the MeSHOPs vitamins, we compare vitamins based on the similarity of the strength of association to biomedical subjects, taking the Euclidean distance of the log of the *p*-values for the shared terms in their MeSHOPs. Co-occurrence is limited to informing about entities that are discussed together in literature, and cannot predict entities that have not yet appeared in the same report. Profile comparison allows any pair of entities to be compared indirectly through their share biomedical terms, with the additional advantage of inherently compensating for differing amounts of literature for each entity. Comparing the results of co-occurrence to the profile comparisons in Additional file 1: Figure S3B reveals that the results of clustering by profile are both similar to the bibliographical co-occurrence, such as Vitamin A clustering with Vitamin D, as well as Pantothenic Acid clustering with Thiamine. Profile similarity cluster however can emphasize different similarities from co-occurrence, such as Niacin being more similar to Pantothenic Acid and Thiamine rather than Biotin, and a similarity in annotations between Vitamin E and Ascorbic Acid. MeSHOPs allow us to analyze a set of biomedical entities to highlight known and expected relationships through strength of co-occurrence in biomedical literature, as well as revealing similarities of annotation profiles.

### Inter-group MeSHOP similarity

To explore the challenges arising with inter-group MeSHOP comparisons, we sought to identify links between a subset of genes and brain disorders. We examined the genes of the Notch, Wnt and Hh signaling pathways, with the list of genes for each pathway extracted from KEGG (accessed June 2011) (See Additional file 1: Figure S4). These signaling pathway genes were grouped using the subset of MeSHOPs involving MeSH terms that are the immediate children of the MeSH term “Brain Diseases”. Clustering using their association to the pathway genes, the “Brain Diseases” are arranged into categories, with “Brain Neoplasms” being the most strongly associated to the genes, with “Hypothalamic Diseases” and “Dementia” also broadly associated. “Brain Injuries”, “Intracranial Hypertension” and “Hydrocephalus” are weakly associated to these genes by MeSHOP comparison. We grouped the pathway genes based on “Brain diseases” subset of their MeSHOPs. Rather than grouping distinctly by pathway, the genes are spread across different clusters. A broad spectrum of the pathway genes strongly associated to “Brain Neoplasms”, with a subset also strongly associated with “Hypothalamic Diseases”. Another distinct set of genes associated to “Cerebellar Diseases” are not associated with the previous two groups (See Additional file 1: Figure S4C). MeSHOPs provide a unique quantitative method of visualizing the gene landscape for a particular topic through the associated MeSH annotations.

## Discussion

MeSHOPs are quantitative annotation profiles based on over-representation analysis of MeSH terms attached to sets of articles, where each set or bibliography is associated to a specific biomedical entity such as a gene, disease or chemical. Conveniently visually depicted as word clouds, a MeSHOP includes both common terms frequently arising in a bibliography and rare concepts that arise more than expected by chance. In this report we demonstrate the capacity of the MeSHOP generation procedure to recover known gene annotations (as curated with Gene Ontology terms), use temporal restrictions to demonstrate how MeSHOPs change over time, and introduce methods for the comparison of MeSHOPs for both intra- and inter-class similarity analyses. MeSHOPs can be expected to be widely used by researchers, as they may be generated for any biomedical entity and provide quantitative annotation without extensive curation.

We anticipate that researchers will be most attracted to the convenient generation of annotation images by converting MeSHOPs to word clouds. Convenient visualization methods in bioinformatics have made substantial impacts on communication, as evident in such methods as sequence logos for motifs [7], circos plots for genomics [8], pip-plots and dotter images [9,10] for sequence alignments, and network diagrams for protein systems [11]. MeSHOPs are likely to provide a similar level of convenience for summarizing complex topics for accelerated interpretation. The use of word clouds, of course, has been extensive, including for the display of gene annotation [12,13]. The key advantage of MeSHOPs is that they draw upon the expert curation underlying MEDLINE.

### Technical challenges

MeSHOPs directly measure the significance of the annotated biomedical topics for a bibliography. The significant terms in a MeSHOP are therefore implicated by co-occurrence (guilt by association). The reliability of such over-representation analysis is dependent on the annotation used to generate the results. MeSH terms and Supplemental MeSH Concepts are annotated to MEDLINE articles by subject area experts to indicate the major and minor topics addressed by an article. There are two caveats to the over-representation analysis. Firstly, a co-occurring MeSH term may not apply to the biomedical topic despite appearing in the same paper. This form of erroneous linkage is mitigated when significant *p*-values are supported by multiple co-occurrences in the bibliography addressing the entity. Secondly, co-occurrence can indicate a negative association, as negative associations are annotated in MeSH if they are an important topic of the paper. However, a negative association is unlikely to provoke substantial further literature support, unless it is of substantial research interest or the result inconclusive, at which point the MeSH term emerges as important to the biomedical topic. Thus it is our expectation that further development of MeSHOPs will need to explore measures of confidence for small bibliographies.

### Related work

The use of statistical tests to assign significance values for annotation terms appearing in a text or across gene annotations has been frequently observed in bioinformatics. We calculate *p*-values using Fisher’s Exact test, which have a specific, well-defined interpretation well-suited for over-representation analysis – the probability that the term would be found as prevalently in an equivalent-sized set of articles drawn uniformly at random from the background set of articles – making it possible to set meaningful confidence thresholds and evaluate the scores. These scores highlight strength of association by correcting for the background frequency of occurrence. Fisher’s Exact Test is commonly used in classic Gene Ontology annotation over-representation tools for gene set analysis such as DAVID [14] and as a measure of over-representation of transcription factor binding sites across a set of genes or sequences [15].

A number of publications have incorporated MeSH terms into the analysis of sets of articles. Many studies have attempted to find common themes for groups of genes arising in experimental studies [5,16-18]. Three papers are more similar to the work described here, although each has distinct characteristics. The LigerCat system was developed to provide a more convenient interface for PubMed searching [6]. The system generates a word cloud for MeSH terms arising in articles reported by an initial user query (which could be a single entity such as a gene or drug). The user can then click on the individual terms within the cloud to restrict results in the PubMed search. Comparisons of MeSH-based gene profiles were performed by Sarkar and Agarwal [19], using hierarchical clustering, but only using profiles composed of binary values (whether a term is present or absent from the profile), where a positive setting was made if there was at least one abstract in which the gene name and assigned MeSH term co-occurred.

Agarwal and Searls describe the use of Fisher’s *p*-value for evaluating the association for the genes present in the articles relating to disease [20]. They combine gene2pubmed, GeneRIF and computationally flag gene names in titles and abstracts of the PubMed entries. The tool gene2mesh [21] provides gene profiles with a universal background. MeSHOPs demonstrate that the same statistical analysis can be applied and visualized for any entity associated to biomedical articles. The Gendoo system [22,23] allows users to see MeSH terms associated with a gene or drug, and provides an information gain score to indicate which genes or drugs are most closely linked to a MeSH term. There is no quantitative profile provided, nor the capacity to perform comparisons of distinct entities.

Analysis of biomedical topics over time has been previously performed by Agarwal and Searls [24], where they examine the progression of individual MeSH terms in biomedical articles and genes over time. The contrast the number of articles published for a given topic against other factors such as relative disease burden, the topic areas for a set of high-tier journals and patent filings, showing the extent of publication growth can identify potentially important areas for research. Rajpal et al. [25] examine the significance of topics related to obesity from 2005-2009. Their trend analysis compares, using Fisher’s Exact Test, the prevalence of biomedical topics and genes in 2005 to their prevalence in 2009. These studies demonstrate the importance and relevance of bibliometric analyses such as MeSHOPs in identifying the focus of existing research.

Other data sources can be analyzed by MeSHOPs. Clinical applications for MeSHOPs are indicated by previous work [26] using electronic health records as an alternative source of annotated biomedical literature. Diagnoses and symptoms from the free-text problem summary lists in the health records are examined to highlight associations to patients. Alternatively, the same methodology used for MeSHOPs could leverage other web services such as RANSUM [27] (which has been expanded to STOP [28]), to investigate over-representation of different ontology terms in datasets available at the National Center for Biomedical Ontology. LePendu et al. [29] demonstrate that GO annotations are a high-quality source of articles linked to genes, and demonstrate over-representation analysis using the Disease Ontology. Good et al. [30] show that Gene Wiki articles are a suitable source of biomedical knowledge that can be automatically annotated with ontology terms.

### Future directions

Many extensions of MeSHOPs remain to be explored. Incorporation of the finer shades of MeSH annotation may be feasible. We describe here the use of the MeSH terms in isolation, however, MeSH terms may be assigned ‘subheadings’ by curators. Such subheadings more specifically specify the context of a MeSH term (e.g. a disease reference may be coupled to “diagnosis” or “therapy”). As well, some MeSH terms are marked as major topics – future analysis could use these more nuanced features to refine the MeSHOP approach.

The organizational structure of the MeSH terms could be better addressed for MeSHOP generation. GO is structured as a directed acyclic graph, thus a term may have multiple parent terms. Grossmann applies a variant of the Fisher’s Exact Test – rather than comparing a term against the background frequency for the class, each term is compared against the frequency of its parental terms using “parent–child-union” and “parent–child-intersection” rules [31]. Future work on how to account for parent–child relationships in the MeSH hierarchy in this vein is thus warranted.

As evident with disease MeSHOPs, there is a positive correlation between the number of articles in a bibliography and the number of over-represented MeSH terms. Improved methods to highlight the most relevant biomedical topics may be required to account for this bias. It may be necessary to cap the size of MeSHOPs, or develop a more Bayesian approach for the statistical measurement of term over-representation that accounts for the number of papers contributing to the profile.

MeSHOPs can be generated using any source for bibliographies. Automated extraction of gene symbols from PubMed abstracts, using technology such as iHOP [32], could supply improved gene bibliographies. Subclasses of MeSHOPs, such as species-specific gene profiles could be generated and compared. A drug MeSHOP could be supplemented with the MeSHOPs of other chemical compounds of the same family.

The quantitative comparison of entities through their MeSHOPs opens the possibility of discovery of novel information. Hierarchical clustering is shown here to group entities with known relationships together, but also provides the opportunity for discovery of new relationships by indirectly linking together entities based on the similarity of their topics. We apply Euclidean distance and complete linkage to perform our hierarchical analysis, methods that could be rapidly computed for our MeSHOP profiles and which have previously successfully applied for other bioinformatics clustering applications involving vectors of continuous data such as gene expression profiles. Other forms of linkage could be applied to emphasize different groupings of entities, and there exist a plethora of similarity measures that could be adapted for comparison of numerical p-value vectors.

## Conclusion

MeSHOPs quantitatively represent the MeSH biomedical terms enriched across a set of papers associated with a specific biomedical entity such as a gene, disease or drug. Visual display of MeSHOPs using word clouds provides a convenient way to convey annotation properties to readers. Comparison between MeSHOPs allows for the generation of hypotheses, opening new avenues for applied text analysis in bioinformatics.

## Competing interests

The authors declare that they have no competing interests.

## Authors’ contribution

All authors contributed to the design of the method and the analysis and interpretation of the data. WAC implemented and carried out the study. All authors read and approved the final manuscript.

## Supplementary Material

Additional file 1 **Are available Online.** Additional files Figures S1-S4 and Additional files Tables S1 and S2. Click here for file
