# Supplementary material for: Quantitative biomedical annotation using medical subject heading over-representation profiles (MeSHOPs)
Source: BMC Bioinformatics. 2012 Sep 27;13:249. doi: 10.1186/1471-2105-13-249 (PMC3564935; doi:10.1186/1471-2105-13-249)
Supplement: Additional file 1 — Are available Online. Additional files Figures S1-S4 and Additional files Tables S1 and S2. [file 1471-2105-13-249-S1.pdf]

SUPPLEMENTAL TABLES AND FIGURES

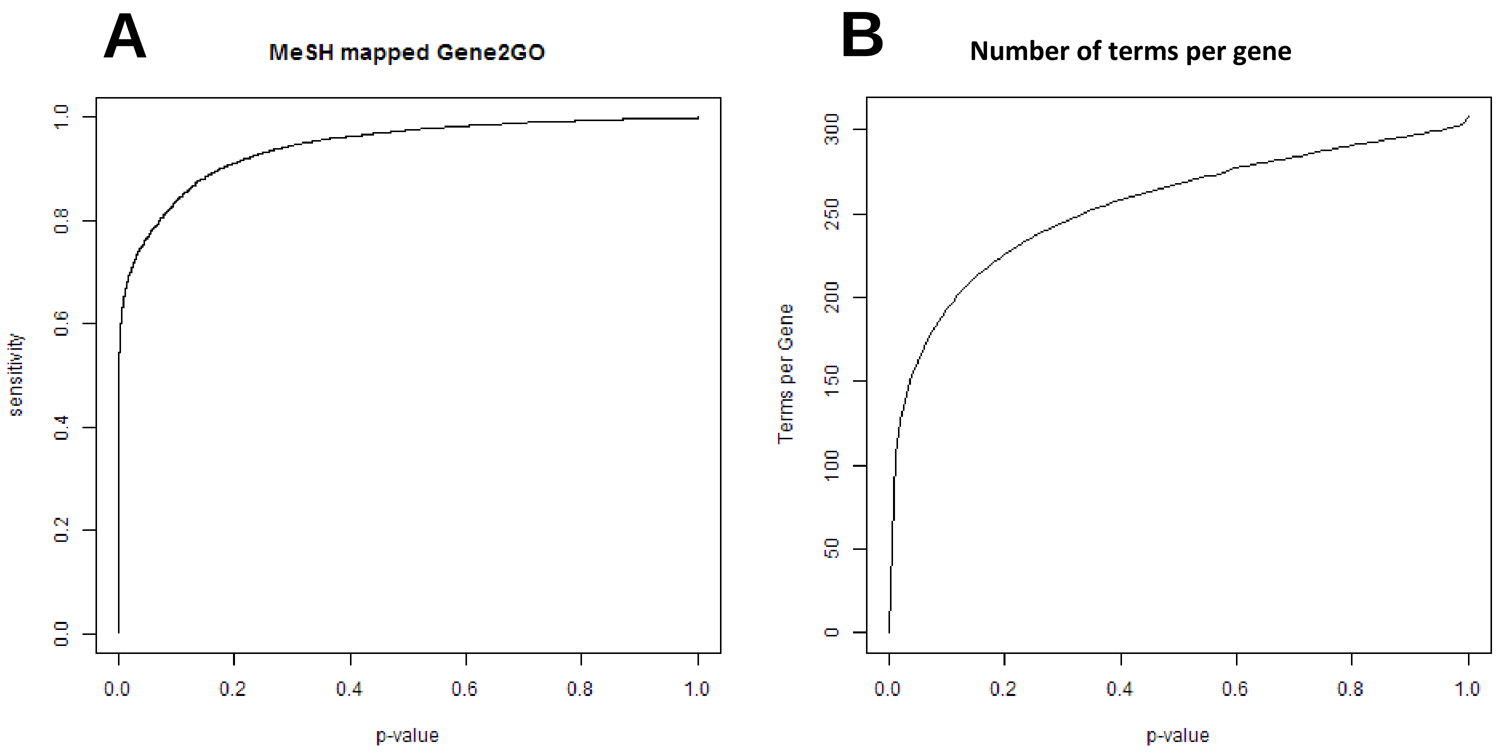

Supp. Fig. 1. *p*-values of MeSH term mapped Gene Ontology (GO) Human Gene annotation. As seen in (A), the majority of human gene-GO annotations were recovered in MeSHOPs with very low p-value scores, indicating that they are very strongly associated in the literature for the genes. (B) The number of MeSH terms per gene plotted against the MeSHOP p-value. The GO terms were mapped to MeSH using UMLS.

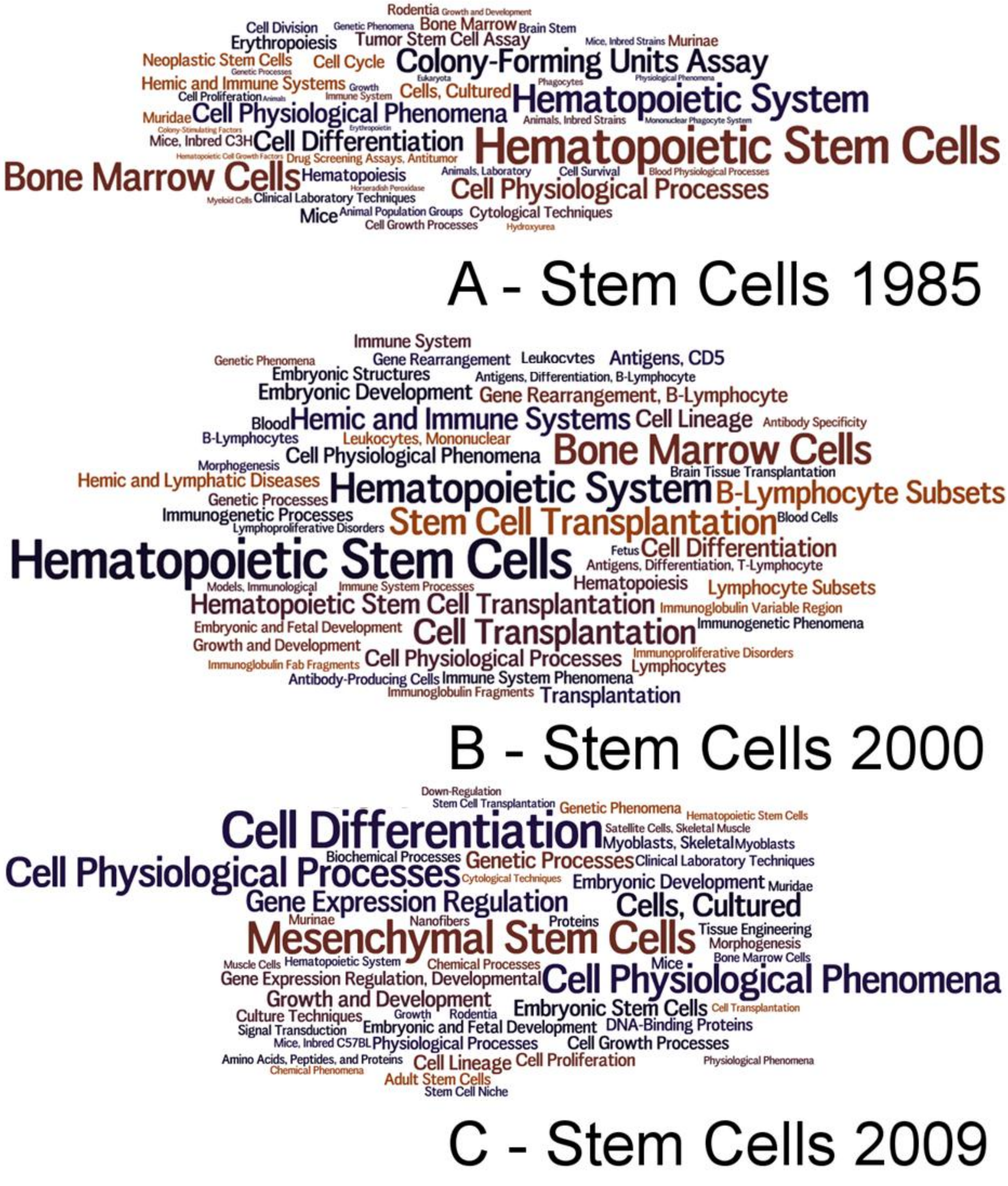

Supp. Fig. 2. MeSHOP for Stem Cells. MeSHOP generated taking the 50 most recent articles annotated with the MeSH term “Stem Cells” from the year 1985 (A), the year 2000 (B) and the year 2009 (C). MeSHOPs were computed using the universal background from PubMed Baseline 2010. The MeSH term for “Stem Cells” and its parent term “Cells” were removed to highlight the other terms present.

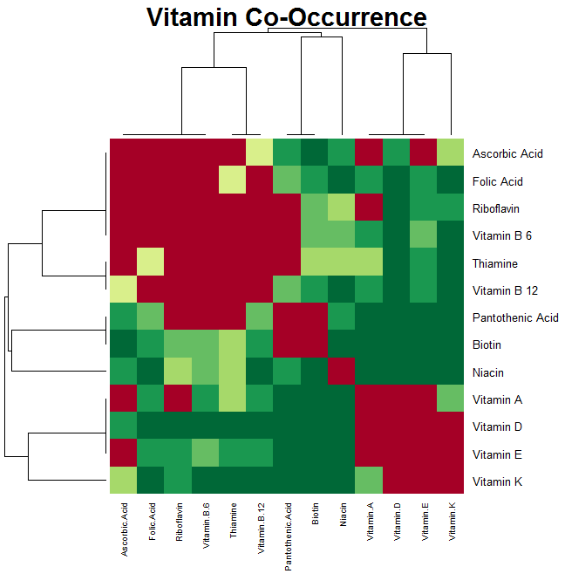

A

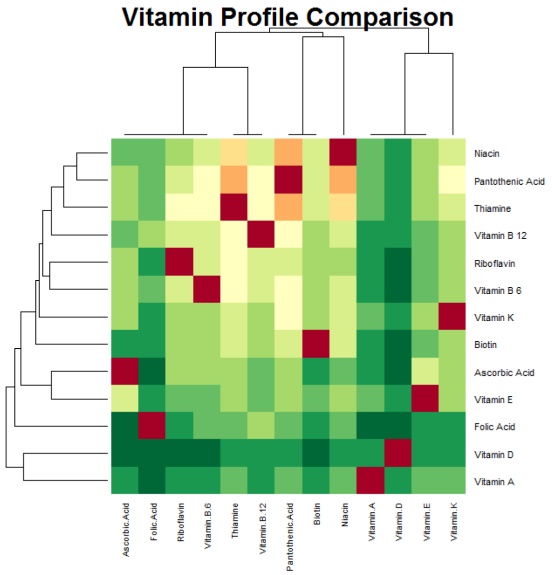

B

Supp. Fig. 3. (A) Co-occurrence of Vitamins through MeSHOPs. Each row represents the MeSHOP for a particular Vitamin. Each column in a row plots the p-value for the MeSH term of the column for the MeSHOP of the row. P-values were computed using the universal Baseline 2010 background. P-values were plotted as a heatmap where red indicates low p-values and green indicates high p-values. The dendrogram was constructed using hierarchical clustering in R.

(B) Vitamins clustered through similarity of MeSHOPs. The MeSHOPs for the Vitamins were compared using Euclidean distance of the log of the p-values for overlapping terms, and the similarity measures were plotted in a heatmap. The resulting similarity scores were clustered, and the dendrogram for the hierarchical clustering plotted on the y-axis. Red indicates low p-values and green indicates high p-values in the heatmap. For comparison, the dendrogram from (A) was plotted on the x-axis.

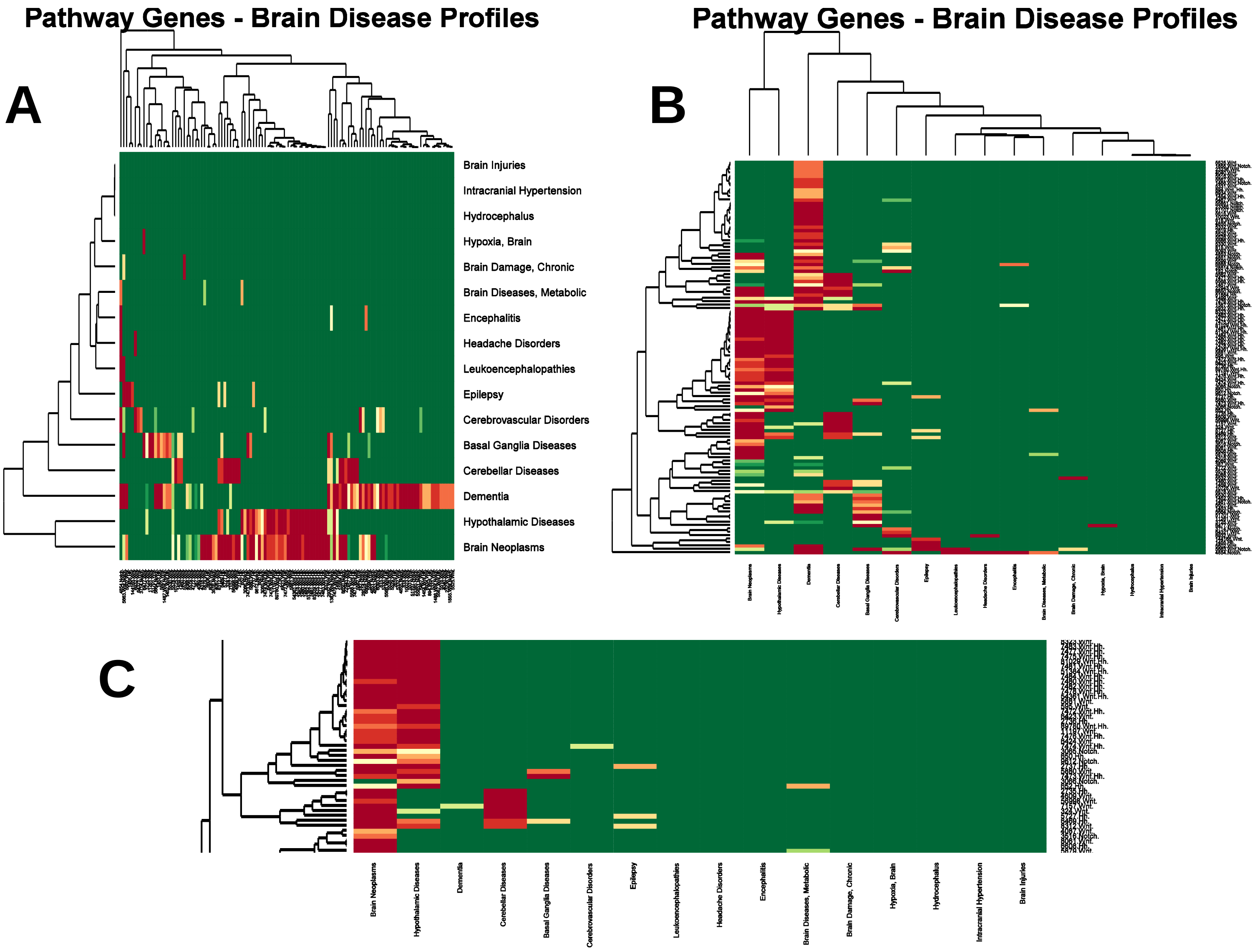

Supp. Fig. 4. (A) Signaling Pathway Gene Co-Occurrence with Brain Disease Annotation. The MeSHOPs for signaling pathway genes from the Notch, Wnt and Hh pathways (columns) were plotted, showing the p-values for their associated Brain Disease MeSH terms (rows). The MeSHOPs were computed using the universal Baseline 2010 background. Hierarchical clustering was performed on each axis, and in the heatmap, red indicates low p-values and green indicates high p-values. (B) Signaling Pathway Gene Co-Occurrence with Brain Disease Annotation. The same data as (A) but with the axes swapped to show more detail of the genes. The MeSHOPs for signaling pathway genes from the Notch, Wnt and Hh pathways (rows) were plotted, showing the p-values for their associated Brain Disease MeSH terms (columns). The MeSHOPs were computed using the universal Baseline 2010 background. Hierarchical clustering was performed on each axis, and in the heatmap, red indicates low p-values and green indicates high p-values. (C) A Subset of the genes from (B) are shown here. Each gene is labeled by their Entrez Gene ID, followed by Wnt, Hh and/or Notch indicating their presence in the respective KEGG pathway.

Dataset		February 2007	January 2009	April 2010
Entrez Gene	**Total Genes**	2 460 748	4 710 910	5 999 558
	**Human Genes**	38 604	40 183	45 423
		**Baseline 2007 (Nov 2006)**	**Baseline 2009 (Nov 2008)**	**Baseline 2010 (Nov 2009)**
PubMed	**Total Articles**	16 120 073	17 764 232	18 502 915
gene2pubmed (Linking Entrez Gene and PubMed)	**Total Links**	3 081 413	12 960 489	5 979 167
	**Total Human Gene Links**	272 123	445 650	527 821

Supp. Table 1. Datasets used in the analysis with details on size and relevant contents. Although the number of human genes has not increased much over the years, the number of non-human links has increased substantially since 2007, while the human gene links have increased at a more moderate rate. Previously, PubMed links from genomic sequence were propagated to all related genes. This practice was discontinued in March 2009, resulting (at the time) in a 60% decrease in links and the disparity in the number of overall links from 2009 to 2010.

	A2M articles	Remainder of PubMed articles	Total
**Articles referring to *Alzheimer Disease***	8	39 265	39 273
**Articles without *Alzheimer Disease* reference**	73	16 080 727	16 080 800
**Total**	81	16 119 992	16 120 073

Supp. Table 2. Analysis of overrepresentation of the MeSH term Alzheimer Disease in the 31 articles linked via GeneRIF to the gene A2M (Entrez Gene ID 2). The raw p-value computed using Fisher's exact test is 1.45E-11, and after Bonferroni multiple testing correction for 25 183 genes, the p-value remains significant at 3.65E-07, indicating a strong research focus of A2M in the field of Alzheimer Disease in existing biomedical literature.

Term	Computational Biology 1999	Computational Biology 2009
Algorithms	NA	8.83E-09
Base Sequence	4.09E-07	NA
Biochemical Phenomena	NA	2.10E-06
Biochemistry	NA	5.00E-21
Biological Evolution	6.16E-09	1.17E-06
Biological Science Disciplines	3.41E-69	1.31E-59
Biology	4.15E-92	2.58E-79
Biomedical Research	3.29E-51	NA
Chemistry	NA	2.27E-18
Chromosomes, Human	1.02E-07	NA
Chromosomes, Human, 21-22 and Y	3.28E-12	NA
Chromosomes, Human, Pair 22	1.42E-14	NA
Chromosomes, Mammalian	1.13E-07	NA
Computational Biology	3.94E-127	1.97E-109
Computer Communication Networks	2.43E-09	NA
Computing Methodologies	4.15E-07	2.92E-11
Databases as Topic	1.52E-21	6.67E-09
Databases, Factual	9.87E-21	1.68E-09
Databases, Genetic	NA	7.25E-10
Electrophoresis, Gel, Two-Dimensional	NA	6.05E-08
Evolution, Molecular	8.01E-10	2.52E-08
Gene Expression Profiling	NA	5.18E-06
Gene Expression Regulation	NA	1.34E-06
Gene Regulatory Networks	NA	3.16E-08
Genes	9.20E-10	NA
Genetic Phenomena	1.24E-24	2.33E-13
Genetic Processes	NA	1.14E-09
Genetic Research	1.03E-94	NA
Genetic Structures	3.35E-21	4.17E-08
Genetic Techniques	5.05E-13	2.51E-09
Genetic Variation	6.05E-11	NA
Genetics	2.74E-59	6.99E-41
Genome	1.17E-19	NA
Genome Components	2.20E-11	NA
Genome, Human	1.97E-12	NA
Genomics	8.54E-76	3.13E-52
High-Throughput Screening Assays	NA	1.14E-06
Human Genome Project	2.69E-100	NA
Information Science	4.08E-28	2.27E-09
Information Services	1.89E-36	NA
Information Storage and Retrieval	1.07E-20	1.63E-08

**Information Systems**	8.92E-18	3.48E-07
**Internet**	4.61E-10	NA
**Investigative Techniques**	NA	5.43E-07
**Medical Informatics**	3.02E-14	8.72E-07
**Medical Informatics Applications**	2.47E-14	7.84E-07
**Metabolomics**	NA	1.52E-09
**Models, Biological**	NA	9.01E-07
**Models, Theoretical**	NA	7.91E-11
**Molecular Structure**	NA	6.18E-06
**Natural Science Disciplines**	9.32E-55	3.42E-47
**Proteome**	NA	1.39E-08
**Proteomics**	NA	2.04E-38
**Public Sector**	1.45E-07	NA
**Research**	5.70E-39	NA
**Science**	2.45E-38	NA
**Sequence Analysis**	3.55E-14	2.89E-06
**Sequence Analysis, DNA**	1.42E-11	NA
**Software**	NA	6.93E-10
**Tandem Mass Spectrometry**	NA	9.89E-07

Supp. Table 3.The top 40 Terms from the MeSHOPs for Computational Biology from 1999 and 2009. Terms are listed in alphabetical order to facilitate comparison between MeSHOPs.
